# Positive effects of reduced tillage intensity on soil organic matter are linked to shifts of *K*-/*r*-strategists in soil bacterial community composition

**DOI:** 10.1093/ismeco/ycag251

**Published:** 2026-09-07

**Authors:** Marie Raab, Jan Helge Behr, Sotirios Vasileiadis, Günter Neumann, Kornelia Smalla, Rita Grosch, Joerg Geistlinger, Wilfried Rozhon, Doreen Babin, Ioannis D Kampouris

**Affiliations:** Department of Agriculture, Ecotrophology, and Landscape Development, Anhalt University of Applied Sciences, 06406 Bernburg, Germany; Plant-Microbe Systems, Leibniz Institute for Horticultural Sciences (IGZ), 14979 Großbeeren, Germany; Department of Biochemistry and Biotechnology, Laboratory of Plant and Environmental Biotechnology, University of Thessaly, 41500 Larissa, Greece; Institute of Crop Science (340h), University of Hohenheim, 70599 Stuttgart, Germany; Julius Kühn Institute (JKI)—Federal Research Centre for Cultivated Plants, Institute for Epidemiology and Pathogen Diagnostics, 38104 Braunschweig, Germany; Plant-Microbe Systems, Leibniz Institute for Horticultural Sciences (IGZ), 14979 Großbeeren, Germany; Department of Agriculture, Ecotrophology, and Landscape Development, Anhalt University of Applied Sciences, 06406 Bernburg, Germany; Department of Agriculture, Ecotrophology, and Landscape Development, Anhalt University of Applied Sciences, 06406 Bernburg, Germany; Julius Kühn Institute (JKI)—Federal Research Centre for Cultivated Plants, Institute for Epidemiology and Pathogen Diagnostics, 38104 Braunschweig, Germany; Julius Kühn Institute (JKI)—Federal Research Centre for Cultivated Plants, Institute for Epidemiology and Pathogen Diagnostics, 38104 Braunschweig, Germany; Department of Applied Microbial Ecology, Helmholtz Centre for Environmental Research GmbH—UFZ, 04318 Leipzig, Germany; German Centre for Integrative Biodiversity Research (iDiv) Halle-Jena-Leipzig, Deutscher Platz 5e, 04103 Leipzig, Germany

**Keywords:** rhizosphere, soil, lifecycle traits, *K-/r*-strategists, microbiome, long-term field experiment, growth rates, soil health, cultivator tillage, plough tillage

## Abstract

Compared to conventional ploughing, reduced tillage intensity (e.g. cultivator tillage) benefits soil organic matter (SOM) accumulation, which has been attributed to lower disruption of soil aggregates under reduced tillage intensity. While soil microorganisms have been known to contribute to SOM accumulation, the contribution of microbial processes to SOM content has been overlooked, with the underlying mechanisms remaining unclear. We here estimated how cultivator tillage shifts soil bacterial community composition toward slower-growing *K*-strategists, in contrast to faster-growing *r*-strategists, by analyzing previously generated data from a long-term field trial in loess chernozem soil. We annotated the 16S rRNA genes from the whole amplicon sequence variant dataset through sequence alignment and identified publicly available genomes phylogenetically related to them with at least 97% sequence identity. Next, we computationally inferred traits such as growth rate, genome size, and the number of carbohydrate catabolism-related genes. These traits were strongly associated with positive responders to cultivator or mouldboard-plough tillage in the bulk soil bacterial communities. Moreover, mouldboard-plough tillage showed a lower ratio of slower- to faster-growing bacterial taxa compared to cultivator tillage in the bulk soil. While this relationship was absent in rhizosphere bacterial communities, these communities showed an overall lower ratio of slower- to faster-growing bacterial taxa than the bulk soil bacterial communities. Moreover, this ratio was negatively correlated with SOM only under mouldboard-plough tillage in bulk soil bacterial communities. Overall, our results indicate that tillage intensity influences the relationship of bacterial *K-*/*r*-strategists with SOM.

## Graphical abstract

**Graphical abstract:**
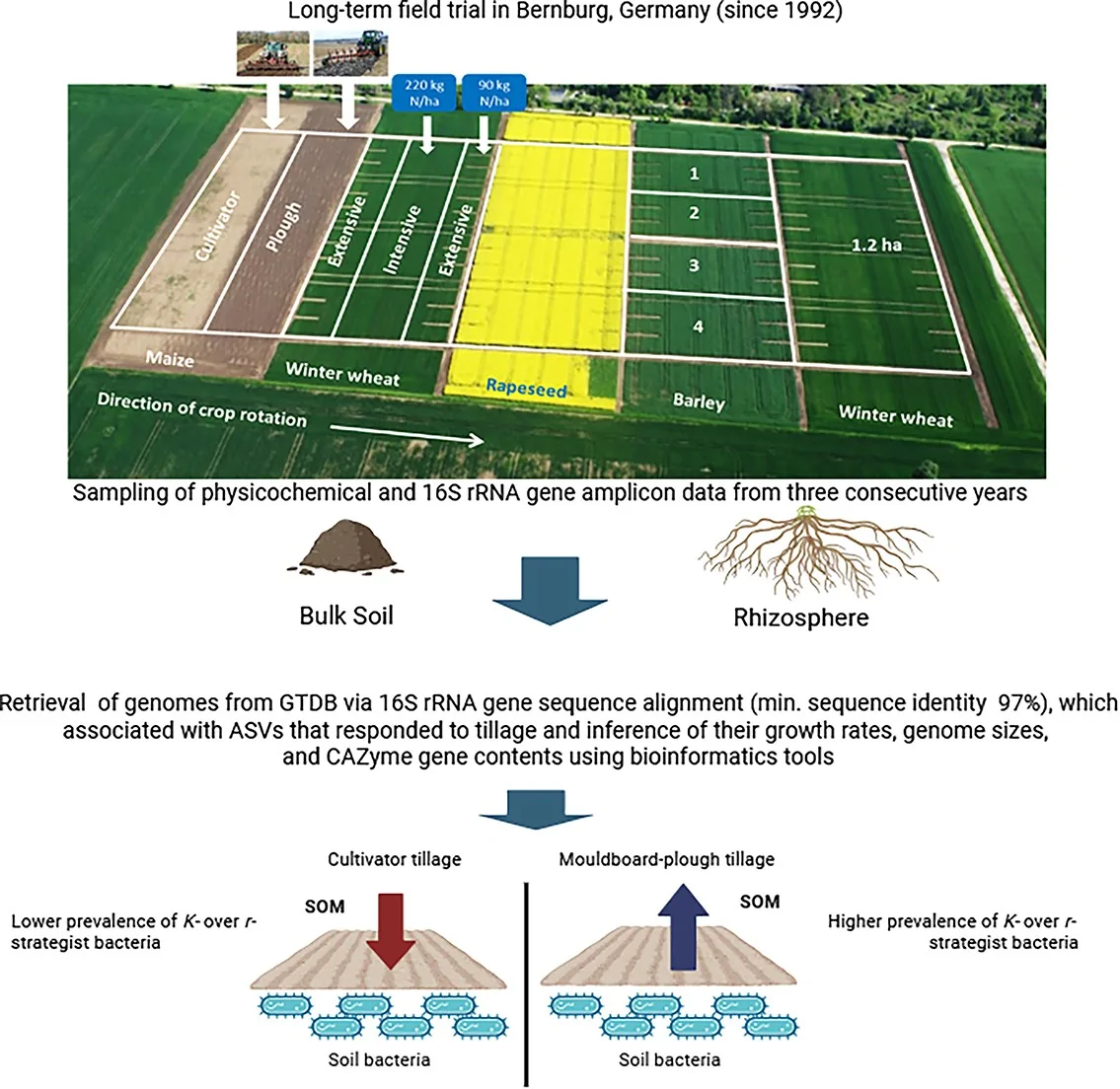

The graphical abstract was created with selected graphical elements from BioRender (https://www.biorender.com).

## Introduction

A healthy soil sustains biological productivity (e.g. nutrient cycling, water regulation, and pest and disease control), maintains environmental stability, and promotes plant, animal, and human health [1]. Intensive agricultural practices compromise soil health [2] and reduce the biodiversity of both soil fauna [3] and microbial communities [2]. It is currently projected that intensive agriculture will ultimately lead to yield losses, mainly due to unsustainable soil management [4]. To counter these negative effects, the adoption of less intensive farming practices, such as conservation agriculture, has been suggested [5]. Conservation agriculture refers to integrated crop production systems based on three principles: minimal soil disturbance, permanent soil cover, and diversified crop rotations [6]. This approach can lead to improvements in soil chemical, physical, and biological properties, such as increased carbon storage and resilience to climate variability [5, 7, 8]. Our previous results from a long-term trial showed significant and stronger effects of tillage practice on soil organic matter (SOM) and total nitrogen (N) content, and the composition of soil prokaryotes than N-fertilization intensity. Specifically, cultivator tillage, which loosens and mixes the soil without inverting it, positively affected plant health and SOM compared to intensive mouldboard-plough tillage, which mixes the soil by inversion [9]. SOM is important for soil fertility, and its maintenance or increase is therefore a major goal for sustainable farming practices. For instance, SOM improves soil aggregates, increases water retention, stabilizes soil pH, chelates metals, provides nutrients, and boosts biological activity [10, 11].

Plant litter and microbial biomass are the major inputs for SOM [12]. While the loss of SOM stocks in agricultural soils is largely due to cropping and the removal of the harvested products from the fields [12], microorganisms have been known to contribute to SOM content [10]. Specifically, microorganisms play a role not only in the decomposition but also in the formation of SOM through excretion from living cells or during necromass formation [13–16]. Thus, soil microorganisms are crucial for soil health [17]. Previous studies proposed that intensive tillage dilutes the SOM in the soil horizon, leading to differences in the stratification of the SOM stocks over depth, where under less intensive tillage, SOM accumulates in the topsoil profile [18, 19]. However, recent studies have argued that ploughing also disrupts soil aggregates, making SOM available for decomposition by soil microorganisms [20]; thus, microbial processes contribute to these SOM differences due to tillage intensity [9, 21–26]. While these processes have been described, an ecological-theoretical framework that addresses how the effects of less-intensive tillage on SOM are connected to shifts in soil microbiota is still lacking.

Ecological theory tends to dichotomize the life strategies of organisms based on specific lifecycle traits, such as specialist/generalist or oligotroph/copiotroph [27]. The copiotroph-oligotroph dichotomy, which is derived from *K-/r*-selection in macroecology, is often used to describe microbial life history theory [28]. Copiotrophic species (*r*-strategists) exhibit faster growth rates, flourish in environments with highly available carbon pools, and have high mineralization rates. Oligotrophs (*K*-strategists), instead, are characterized by slower growth rates and are adapted to compete and survive when resources are limited [29]. Related traits, such as microbial growth rate, genome size, and carbohydrate metabolism, can be used to elucidate relationships between environmental parameters and microbial life strategies. For instance, genome size was shown to be a strong predictor of soluble organic matter and functional molecular diversity [30]. Using soil metagenomes, Osburn *et al.* [31] demonstrated that a high bacterial growth rate potential was associated with highly productive forest ecosystems and established a microbial physiological framework to better understand soil carbon cycling. Less intensive farming practices, such as cultivator tillage, intercropping (e.g. cereal with legume plants), and wide crop rotation, often with the integration of cover crops, are hypothesized to increase SOM by stimulating microbial processes [2, 32]. However, it remains unclear how the proposed microbial processes are connected to different microbial life strategies, and specifically, whether the positive effects of less intensive tillage on SOM are connected to the dominance of *K*- or *r-*strategists.

In the present study, we aimed to investigate how tillage intensity affects the composition of bacterial communities in relation to the abundance of *K*- and *r*-strategist taxa, by using data generated from our previous studies in a long-term field experiment (LTE) in Bernburg, Germany [9, 33]. The fields at the LTE site contain plots managed with mouldboard-plough or cultivator tillage and, each with conventional (intensive) or 50% reduced (extensive) N-fertilization since 1992 [34]. Microbial data generated from these experiments, along with soil and plant physicochemical data from the LTE site, were deposited in a public data repository [33, 35]. By analyzing data obtained across three years, we estimated how farming practices shaped soil characteristics and bacterial communities, using maize as a model crop (sampled in the years 2019, 2020, and 2021). In addition, amplicon sequence variants (ASVs) were mapped to the 16S rRNA genes of genomes retrieved from the Genome Taxonomy Database (GTDB) [36], with sequence alignment and the closest genomes (>97% sequence homology). Using these genomes, the growth rates were predicted based on codon usage and genome sizes, and the number of CAZyme genes (carbohydrate-active enzymes) per genome was estimated [37]. Then we estimated whether the ASVs that responded to tillage practices showed higher or lower values for each trait. Finally, assigned slower- and faster-growing bacteria based on the distribution and the median values of growth rates for the total population of mapped ASVs, as an indication of how tillage influences the compositional ratio of *K-*/*r*-strategists, in relation to the SOM content.

## Materials and methods

### Field trial description and sampling

Soil samples to a depth of 30 cm were taken for physicochemical analysis from a maize field (*Zea mays* L. cv. Benedictio, KWS Saat SE & Co. KGaA) grown in the long-term field trial (LTE) in Bernburg, Germany (51° 82′ N, 11° 70′ E). The crops followed a rotation cycle with winter rapeseed (*Brassica napus* L. cv. Florida), winter barley (*Hordeum vulgare* L. cv. Higgins), winter wheat (*Triticum aestivum* L. cv. Lemmy), grain maize (*Z. mays* L. cv. Benedicto), and again winter wheat (cv. Lemmy). The same plant varieties were used throughout the three years. The soil of the field site is characterized as loess chernozem over limestone (8% sand, 70% silt, 22% clay; pH 7.0–7.4) as described in detail by Sommermann *et al.* [34]. The respective field plots (1.2 ha, subdivided into four replicates) contained two areas of tillage practices: mouldboard-plough (MP, 20–30 cm depth, soil inversion) and cultivator tillage (CT, 12–15 cm depth, soil loosening). These strips were subdivided into strips with standard N-fertilization intensity (2019: 100 kg N ha^−1^, 2020: 120 kg N ha^−1^, 2021: 120 kg N ha^−1^), including the application of pesticides (intensive, Int) and a reduced N-fertilization intensity (2019–2021: 40 kg N ha^−1^) without fungicide and growth regulator use (extensive, Ext). Winter wheat was the pre-crop of maize in all three years. The field experiment was managed with four treatments (MP-Ext, MP-Int, CT-Ext, and CT-Int), each in four replicates. As previously described [9], soil and maize rhizosphere samples were collected at flowering (EC 53–63) during three growing seasons from 2019 to 2021 (please check the Supplementary Material Information for further details). DNA was isolated from 0.5 g of bulk soil (BS) as previously described [38], and rhizosphere (RH) DNA was extracted from 0.5 g of soil detached from 5 g of roots [39]. SOM and total N content were analyzed by elemental analysis using a CN828 analyzer (LECO, St. Joseph, MI, USA). SOM was calculated as the difference between total organic matter and carbonate carbon. All data are publicly available as previously described [35].

### Physicochemical data analysis

Physicochemical data were retrieved from our BonaRes repository [33, 35] using customized Python scripts. Data analysis was executed using the R programming language v. 4.2 [40]. For all physicochemical data analyses, general R packages were applied: “ggplot2” v. 3.5.1 [41] and “ggpubr” v. 0.6 [42] for graphs and the Wilcoxon rank-sum test with Benjamini–Hochberg correction for each year. For data handling, we used “tidyverse” v. 2.0 [43], “dplyr” v. 1.1.4 [44], “reshape2” v. 1.4.4 [45], “stringr” v. 1.3.1 [46], and “parallel” v. 4.4.2 [47]. SOM and total N were analyzed with ANOVA and Tukey’s HSD test and linear models (LM) using the package “car” v. 1.1.36 [48]. Data were standardized (z-score transformation), resulting in a mean of 0 and a standard deviation of 1. Subsequently, the models were applied to estimate the standardized model coefficient (β_1_-coefficient), which indicates the effect size of the treatments. We applied parametric bootstrapping to estimate *P*-values using a two-sided sign-based bootstrap approach (10 000 iterations). Additionally, 95% confidence intervals (CI) and median coefficient values inferred from 10 000 bootstraps are provided for each β_1_-coefficient. Significance was set at *α* = 0.05, and the effects of treatments were described based on tillage, N-fertilization intensity, and year: y ~ Tillage intensity × N-fertilization intensity × Year. In addition, the 95% CIs for the standardized model coefficients indicated significant differences from the null model, as well. Data were checked for normality and homogeneity of residuals (Fig. S1). A negative β_1_-coefficient indicates a positive impact on the variables under less intensive soil and N-fertilization management (cultivator tillage or extensive N-fertilization), whereas a positive β_1_-coefficient indicates a positive effect under more intensive practices (mouldboard-plough tillage or intensive N-fertilization). In addition, a trend analysis with the data from a previously available time-point of the LTE (2009) was performed to show the increase of SOM and N over the years (Fig. S2).

### Bioinformatics and statistical analysis of amplicon sequencing data

#### 16S rRNA gene amplicon sequencing

16S rRNA gene sequencing library construction was carried out at two different facilities across the different studies, with slight modifications of the same primer pairs. Specifically, sequencing was performed using Illumina MiSeq and the primers Uni341F (5′-CCTAYGGGRBGCASCAG-3′) and Uni806R (5′-GGACTACHVGGGTWTCTAAT-3′) in 2019. PCR amplification of the V3–V4 region of the 16S rRNA gene was performed as previously described [49]. Sequencing was carried out on an Illumina MiSeq platform using Reagent Kit v2 (2 × 250 bp; Illumina, San Diego, USA) following the manufacturer’s instructions. Meanwhile, for samples from 2020 and 2021, sequencing was carried out by Novogene (Cambridge, UK) on NovaSeq 6000 PE250 using the primer Uni341F and a modified version of the primer 806R (5′- GGACTACNNGGGTATCTAAT-3′), and analysis of 16S rRNA amplicon sequences was performed as previously described [50]. Adequate sequencing depth was estimated using rarefaction (Fig. S3). ASV abundance was normalized to the sample with the lowest number of reads (21701 reads) via rarefaction, which was repeated 1000 times, and the average read abundances were calculated.

#### Differential abundance testing and multivariate tests

For differential abundance analysis of the microbiome, we performed logistic regression (LR) with Benjamini–Hochberg correction in the R programming language for each year separately. ASV responders with significantly higher fold-change (FC) under cultivator or mouldboard-plough tillage were considered as positively associated with either cultivator (CT-responders) or mouldboard-plough (MP-responders) tillage (LR, *P* < .05). To link the cumulative relative abundances of ASVs that positively responded to cultivator tillage with SOM, we performed LMs with 10 000 bootstraps as described above. The significance of tillage and N-fertilization intensity on community composition was tested with PERMANOVA based on the package “vegan” v. 2.7.2 [51].

### Retrieving genomes from the Genome Taxonomy Database phylogenetically associated with differentially abundant bacterial ASVs

Closest phylogenetically associated genomes to all ASVs were retrieved from the GTDB according to ASV alignments and selected for estimating three different traits in bacterial genomes that associate with *K*- or *r*-strategies: minimal doubling time, genome size, and carbohydrate metabolism. Since the differences in lifecycle traits among species of the same genus are negligible [52, 53], we selected the genus level for trait estimation. However, because (i) taxonomic classification is arbitrary for several taxa, (ii) several potential mismatches between SILVA and GTDB exist, and (iii) the Naïve Bayes classifier does not usually classify ASVs at the genus level in several cases, we selected an alignment-based approach. Thus, ASVs were aligned to the 16S rRNA gene dataset from the GTDB using the USEARCH global alignment tool [54] with a minimum sequence similarity of 97%. We selected the 97% threshold for accounting for potential PCR and sequencing errors while estimating the closest relatives at the genus level, since the size of the amplified region confers sufficient resolution for genus-level estimation [55]. The genomes, whose 16S rRNA gene sequences were annotated to ASVs, were retrieved from the SRA (sequence read archive, www.ncbi.nlm.nih.gov/sra) using EMBOSS toolsets v. 6.6.0 [56].

### Estimation of traits associated with *K-*/*r-*strategists in bacterial genomes

Predicted minimal doubling times (pmDTs) were inferred based on codon usage bias via gRODON2 v. 2.4.0 [37]. Genome sizes were predicted by CheckM2 v. 1.1.0 [57] (Supplementary Material). In addition, the number of genes for carbohydrate-active enzymes (CAZyme genes) per genome was annotated using PROKKA v. 1.14.5 [58], and subsequent gene annotation was performed with DIAMOND blastx (command options: -id 0.8 --query-cover 0.4, v. 2.1.8) [59] on the predicted open reading frames using the dbCAN3 database [60]. To estimate differences in genome size and doubling time between genomes associated with ASVs enriched in cultivator and mouldboard-plough tillage, we performed Wilcoxon rank-sum tests in R. Since mapping ASVs to publicly available genomes harbors inherent issues, e.g. one ASV may be mapped to more than one genome, or ASVs might belong to taxa without a close phylogenetic genome representative in the databases, we accounted for these issues using a distribution-wise method. We first assigned each mapped ASV a trait-value (pmDT, genome size, CAZYme genes per genome) based on the mean values of aligned genomes. Then we inferred their distribution, divided the population based on the median values, and we computed a ratio for each trait (e.g. faster-growing taxa < median pmDT and slower-growing taxa > median pmDT, Fig. S4). These ratios served as a comparative indication that each treatment might compositionally select for specific traits in the whole bacterial communities, rather than for specific taxa, e.g. for faster- against slower-growing bacterial taxa. We tested how the ratio of relative abundance between above/below median values differed from each treatment and soil habitat using the Wilcoxon rank-sum test and how it correlated with SOM, using Spearman’s correlation and polynomial linear models for non-monotonic relationships (for further details, please check the Supplementary information).

## Results

### Cultivator tillage strongly promoted soil organic matter and total nitrogen content across different years and shaped the bacterial community composition

To estimate the effects of tillage on SOM and total N content, we analyzed three years of data from a long-term experimental field site under different N-fertilization intensities. Cultivator tillage, the less intensive tillage management, consistently showed higher SOM and total N content than mouldboard-plough tillage for all three years (Wilcoxon test, *P* < .05, *n* = 4). By comparing data from 2009, we found that both SOM and N had lower values than in 2019–2021, with cultivator tillage showing slightly higher rates of SOM and N accumulation than mouldboard-plough tillage (Fig. S2). Using a global LM across all three years (2019–2021), we found that tillage significantly affected SOM and total N content in the soil, whereas year, as a variable, increased N content (LM, *P* < .05; Fig. 1; Table S1). Consequently, our results indicate that cultivator tillage led to higher accumulation of SOM and N content in the soil across all years. Moreover, tillage had a strong and significant effect on bacterial community composition across all three years in both soil habitats (PERMANOVA, *R*^2^ = 12.2%–30.2%, *P* < .05, *n* = 16, Fig. S5), whereas the effect of N-fertilization intensity was weaker and not consistently significant across all years. Thus, tillage strongly shaped not only SOM and N content but also bacterial community composition, whereas N-fertilization intensity effect was weak and often not significant.

**Figure 1:**
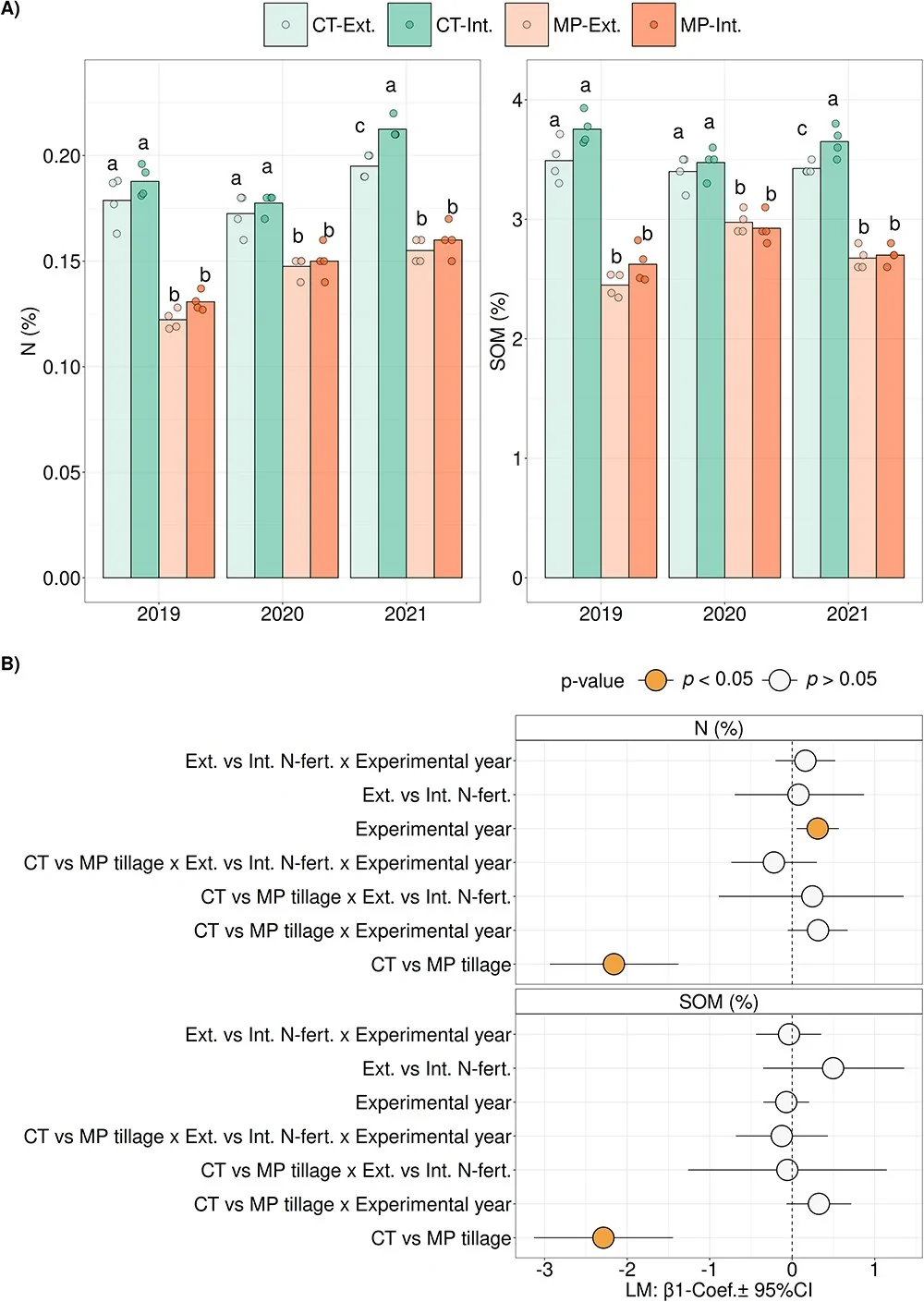
Tillage and N-fertilization effects on total nitrogen (N) and soil organic matter (SOM). (A) SOM and N content from the long-term experimental field site, sampled in 2019–2021, under different tillage intensities (cultivator (green) vs. mouldboard-plough (orange)) and N-fertilization intensities (extensive (light transparency) vs. intensive (dark transparency), *n* = 4). Different letters indicate significant differences at *P* < .05 according to Tukey’s honest significant differences (HSD) post hoc test. The analysis was performed after ANOVA to control the Type I error rate across multiple comparisons. (B) Standardized (β_1_) coefficients from linear models on SOM and total N content with tillage, N-fertilization intensity, and year as fixed effects. Each dot represents the mean estimated β_1_-coefficient of the fixed effects, inferred from 10 000 bootstraps. Error bars indicate the upper and lower 95% CIs. The color scale indicates the *P*-values, derived from bootstrapping. Generally, β_1_-coefficients above 0.5 or below −0.5 were considered an indication of a strong effect size. CT: cultivator, MP: mouldboard-plough, Int.: intensive, Ext: extensive, LM: linear model, Coef: coefficient, CI: confidence interval.

### Differentially abundant ASVs responded to tillage intensity in bulk soil and rhizosphere

To gain further insights into bacterial ASV responders positively associated with either cultivator (CT-responders) or mouldboard-plough (MP-responders) tillage across each analyzed growing season, we performed differential abundance analysis (LR, *P* < .05, Fig. 2, Table S2). In 2019, 35 ASVs were significantly enriched by cultivator tillage in both soil habitats (BS and RH), whereas only five ASVs were enriched in 2020 and just two ASVs in 2021 (*P* < .05, Fig. S6). Additionally, higher relative abundance due to mouldboard-plough tillage was observed in the rhizosphere only in 2021 (18 vs. 13 ASVs, *P* < .05, Fig. S6). Furthermore, 37 ASVs were enriched in mouldboard-plough tillage in both soil habitats in 2019, while in 2020, there were five ASVs, and in 2021, three ASVs. Overall, the number of common ASVs in both soil habitats was higher in mouldboard-plough tillage than in cultivator tillage. Furthermore, we identified three ASVs in the bulk soil that showed consistent responses over all three years (Fig. S7). One MP-responder was identified at the lowest taxonomic level as Xanthomonadales (ASV 526), while two CT-responders were classified as Cytophagales (ASV172 and ASV1370) (Table S2). In contrast, no consistent responses were observed in the rhizosphere across all years. Since the differential abundance plots indicated that tillage primarily affects the dominant taxa, we performed β-diversity analysis again using the top 50 most abundant taxa per year, which confirmed that tillage strongly affects the dominant members of the community (PERMANOVA, *R*^2^ = 18.8–46.1%, Fig. S5).

**Figure 2:**
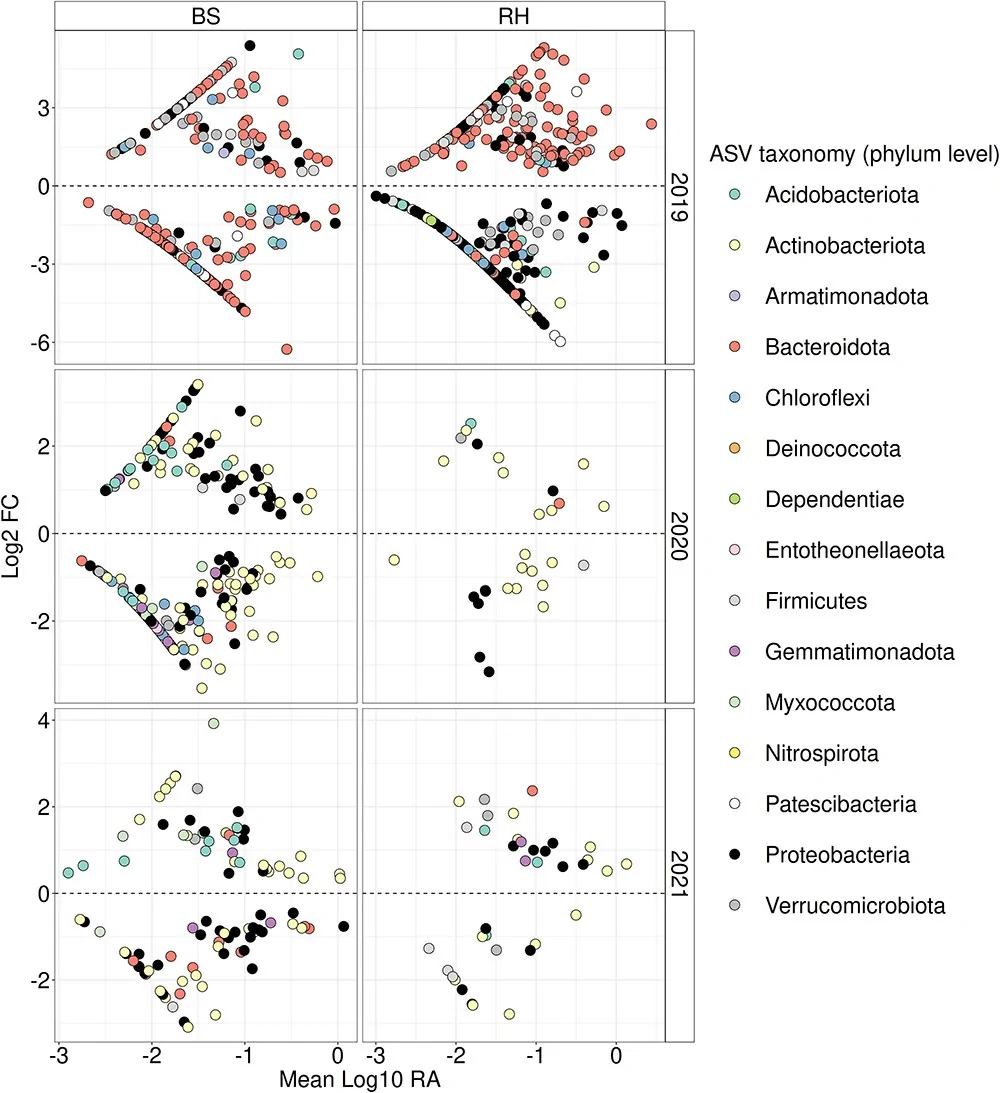
Differential abundance of bacterial ASVs due to tillage intensity in different years and soil habitats. The significant differences were estimated from a logistic regression on centered log-ratio-transformed relative abundances (to account for compositionality) and Benjamini–Hochberg correction (adj. *P* < .05, *n* = 4). Each dot represents a responder ASV, based on the log_2_ fold change (log_2_FC) from less-intensive cultivator tillage (negative) to intensive mouldboard-plough tillage (positive) and plotted against the mean relative abundance (mean Log_10_ RA). Dots are color-coded by phylum, and the data are partitioned by soil habitat (rhizosphere (RH) or bulk soil (BS)) and year to highlight differences in relative abundance (RA) patterns per individual habitat and year. Models were fitted separately for each year and soil habitat.

### Responder ASVs to tillage strongly correlated with soil organic matter

Following the identification of bacterial ASV responders to tillage intensity in bulk soil and the rhizosphere of maize, we evaluated the correlations of these responders with SOM. Thus, we performed linear regressions between SOM and the cumulative relative abundance of bacterial ASVs that were CT- or MP-responders for each soil habitat (BS or RH) across each experimental year. In general, in both soil habitats, the cumulative relative abundance of CT-responders strongly and positively correlated with SOM (LM, bootstrapped β_1_-coef. > 0.73, *P* < .0001, Fig. 3), while the cumulative relative abundance of MP-responders strongly and negatively correlated with SOM (LM, bootstrapped β_1_-coef. < −0.77, *P* < .0001, Fig. 3). Consequently, the relative abundance of responders to tillage strongly correlated with SOM content, independent of the soil habitat.

**Figure 3:**
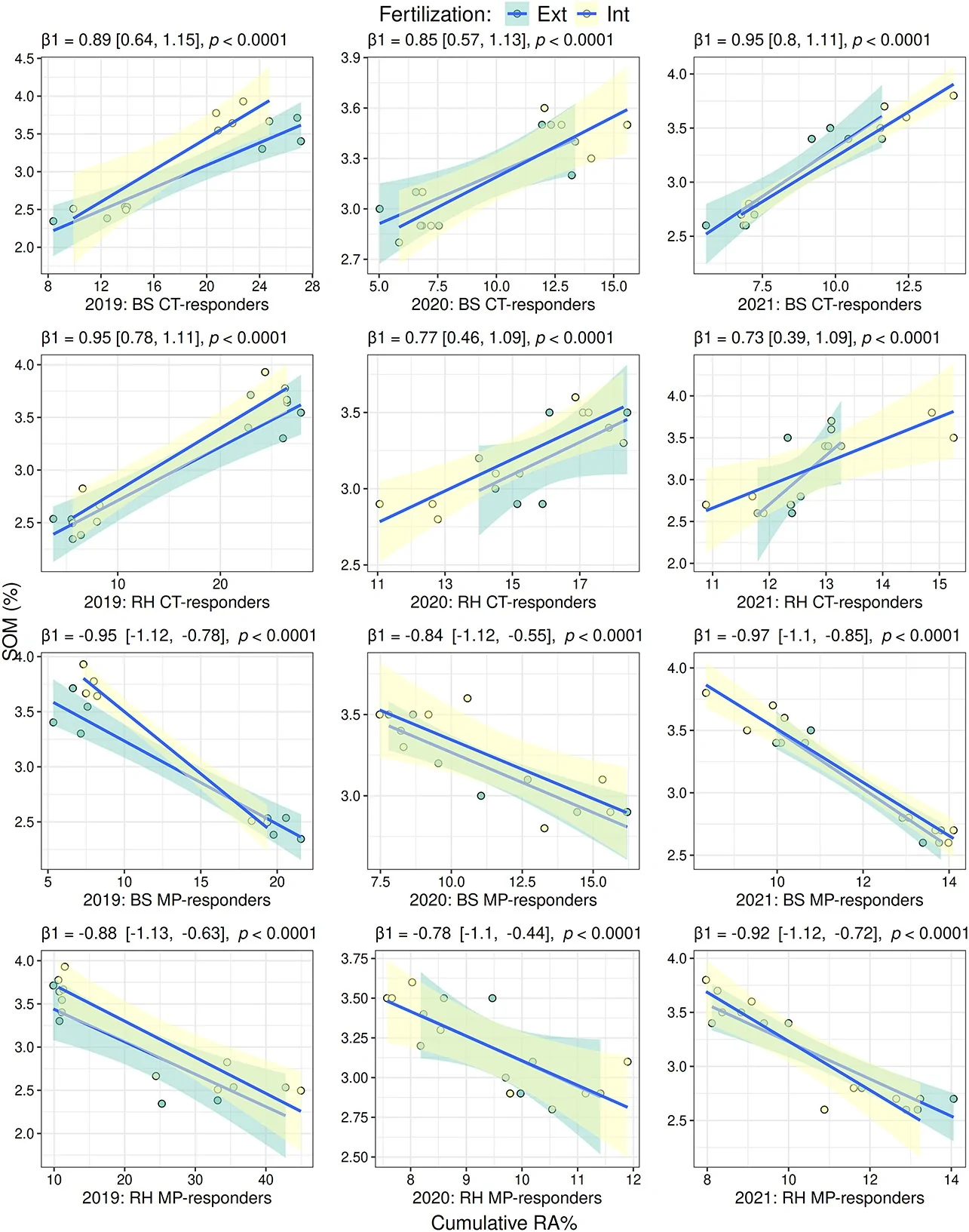
Linear regressions between SOM and cumulative relative abundance (RA%) of bacterial ASVs that were significantly enriched in cultivator (CT-responders) or mouldboard-plough tillage (MP-responders) in the two soil habitats, bulk soil (BS) and rhizosphere (RH), for each year. Smoothed lines indicate the 95% CIs. The scaled β_1_-coefficient, the lower and upper values of the CI, and the *P*-values were inferred from 10 000 bootstraps. The models were fitted separately for each year and habitat. The regression points are colored per N-fertilization intensity.

### Genomes phylogenetically associated with ASV responders to cultivator tillage showed higher minimal doubling times, smaller genome sizes, and fewer CAZyme genes per genome

To gain further insights into whether tillage intensity shaped the compositional proportion of *K*- and *r*-strategists in the soil and rhizosphere bacterial communities, we analyzed three functional traits associated with *K-/r*-strategies for publicly available genomes phylogenetically associated with CT- or MP-responder ASVs based on sequence homology (Table S3). The analyses were performed for all years combined and each year separately. In general, genomes associated with CT-responders in bulk soil showed higher pmDTs when compared to genomes associated with the respective MP-responders (Wilcoxon rank-sum test, *P* < .0001, Fig. 4A). In the rhizosphere, the pmDTs had no significance in 2020 and showed lower pmDTs for CT-responders compared to MP-responders in 2019. All years together presented higher pmDTs for the genomes associated with CT-responders. Overall, genomes phylogenetically associated with CT-responders showed smaller genome sizes than genomes associated with MP-responders in the bulk soil (Wilcoxon rank-sum test, *P* < .0001, Fig. 4B), except in 2021. However, from 2019 to 2021, genome sizes showed an opposite trend for bacterial genomes associated with CT/MP-responders in the rhizosphere. Additionally, genomes associated with CT-responders showed a lower number of CAZyme genes per genome than genomes associated with MP-responder ASVs in the bulk soil (Wilcoxon rank-sum test, *P* < .001, Fig. 4C). In contrast, this trend was reversed in the rhizosphere, where genomes phylogenetically associated with CT-responders showed higher numbers of CAZyme genes per genome than the genomes associated with MP-responders (Fig. 4C).

**Figure 4:**
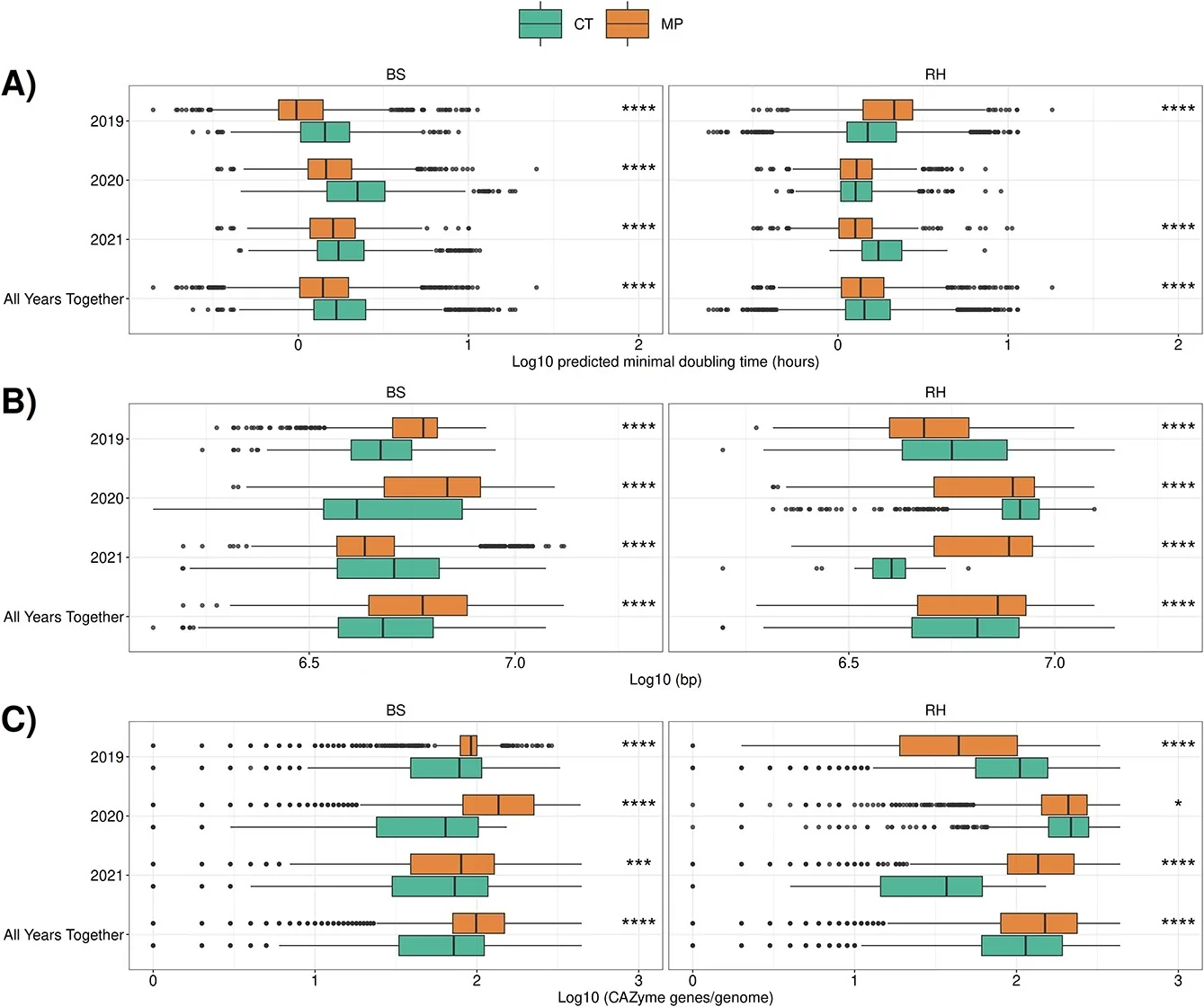
Boxplots showing different traits of bacterial genomes that were phylogenetically related to MP- or CT-responder ASVs: (A) pmDT (hours), (B) genome size (bp), and (C) the number of genes for carbohydrate-active enzymes (CAZyme) per genome. The graph shows only genomes that had high sequence homology (97% alignment identity) to ASVs significantly enriched in one of the two tillage practices: Cultivator tillage (CT), represented in green, and mouldboard-plough tillage (MP), shown in orange. Values were log_10_-transformed to improve visualization. Data are shown for each group of responders separately for bulk soil (BS) and rhizosphere (RH). In addition, we show data for responder ASVs for all years combined as well as for each individual year (2019, 2020, and 2021). Statistical significance was evaluated using the Wilcoxon rank-sum test (^*^P < .05, ^**^P < .01, ^***^P < .001, ^****^P < .0001). Global alignment was performed with ASVs and 16S rRNA genes from the GTDB. Genome size was predicted with CheckM2 and was corrected for completeness and contamination. Minimal doubling times were inferred from codon usage with gRodon2. CAZyme genes were annotated using the dbCAN3 database. BS: bulk soil, RH: rhizosphere.

### Tillage intensity did not consistently influence the proportion of *K-*/*r*-strategist traits in responders belonging to different bacterial phyla

While cultivator tillage clearly enriched the proportion of *K-*strategist taxa in bulk soil bacterial communities when compared to mouldboard-plough tillage (see previous section), it was still unclear whether this effect remained consistent across different taxonomic groups or was caused by shifts toward certain taxonomic groups. To investigate this, we selected the bacterial phyla Actinobacteriota, Firmicutes, and Proteobacteria for further analysis due to a sufficient number of genomes for comparison. Among the pmDT, Proteobacteria showed a strong statistical significance (Wilcoxon rank-sum test, *P* < .0001, Fig. 5A) with genomes phylogenetically associated with CT-responders having longer pmDTs than those associated with MP-responders. Actinobacteria displayed a similar pattern with weaker statistical significance (Wilcoxon rank-sum test, *P* < .01, Fig. 5A). Firmicutes showed no statistical significance. Examining the genome sizes, a strong statistical significance was observed only in Actinobacteriota, which were smaller when phylogenetically associated with CT-responders compared to MP-responders (Wilcoxon rank-sum test, *P* < .0001, Fig. 5B). In contrast, no significant difference was found for Firmicutes and Proteobacteria. Regarding the number of CAZyme genes per genome, significant differences were noted across all phyla, with genomes phylogenetically associated with CT-responders having fewer CAZyme genes than those associated with MP-responders. The significance was stronger for Actinobacteriota and Proteobacteria (Wilcoxon rank-sum test, *P* < .0001, Fig. 5C) compared to Firmicutes (Wilcoxon rank-sum test, *P* < .05, Fig. 5C).

**Figure 5:**
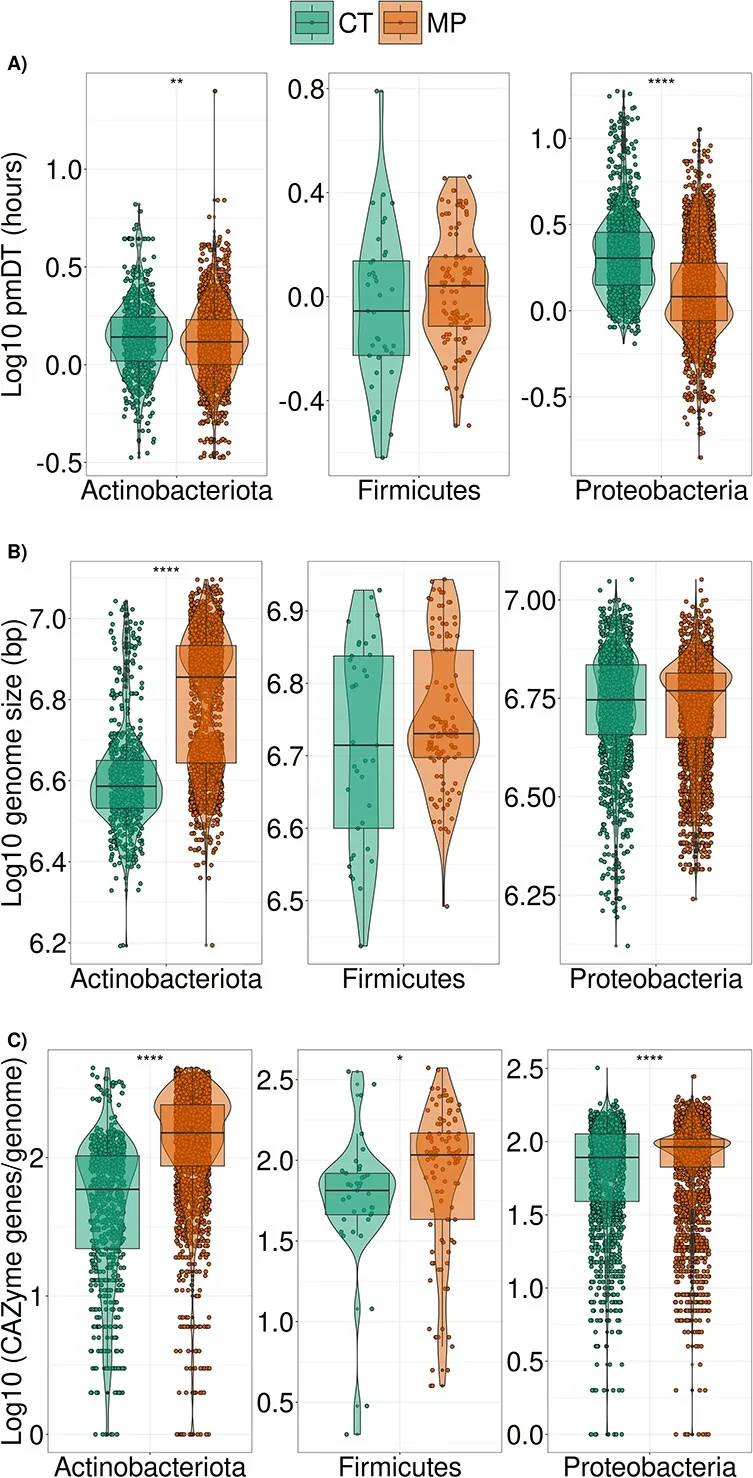
Box and violin plots were based on genomes that showed high sequence homology to ASVs significantly enriched in both tillage practices in the bulk soil and grouped at the phylum level: cultivator tillage (CT), represented in green, and mouldboard-plough tillage (MP), shown in orange. The figure shows phylum-level comparisons for the selected bacterial phyla Actinobacteriota, Firmicutes, and Proteobacteria, since their number of genomes was sufficient for differential testing. (A) pmDT (hours), (B) predicted genome size (bp), and (C) predicted number of CAZyme genes per genome. Values were log_10_-transformed to improve visualization. Statistical significance was evaluated using the Wilcoxon rank-sum test (^*^P < .05, ^**^P < .01, ^***^P < .001, ^****^P < .0001). Genome alignment was performed with ASVs, and 16S rRNA genes were extracted from the global taxonomy database. Only genomes that showed at the 16S locus at least 97% alignment identity with ASVs were included in the analysis. Minimal doubling times were inferred from codon usage with gRodon2. CAZyme genes were annotated using the dbCAN3 database.

### The ratio of slower/faster-growing bacterial taxa increased in response to cultivator tillage in bulk soil bacterial communities

To further verify how tillage is associated with *K-/r-*strategist selection, we aimed to compare the overall compositional ratio of *K-/r-*strategist taxa for each trait for all ASVs with a close genome representative (Fig. S4). Based on the distribution of pmDTs, we identified that all ASV taxa with a close genome representative showed a near-Gaussian distribution with a median of approximately 2.5 hours, after log_10_ transformation, indicating that our analysis mapped the ASV population to a sufficient extent regarding the pmDT (Fig. S4). The ratio of slower- to faster-growing taxa was higher in bulk soil bacterial communities exposed to cultivator tillage (Wilcoxon test, *P* < .05, Fig. 6A). Rhizosphere bacterial communities showed significantly lower *K-/r-*strategist ratio than bulk soil (Wilcoxon test, *P* > .05, Fig. 6A), but their overall ratios did not differ in response to tillage. To further elucidate how the ratio of slower- to faster-growing bacterial taxa correlates with SOM, we performed additional analysis. Overall, the ratio of slower/faster bacterial taxa in bulk soil bacterial communities was positively yet weakly correlated with SOM (Spearman’s correlation, *ρ* = 0.33, *P =* .023, Fig. S8). A polynomial model showed a better fit of the data, suggesting a non-monotonic relationship (*P* = .027, Fig. S8). However, when we divided the two groups (cultivator and mouldboard-plough tillage), we observed a stronger negative Spearman’s correlation (ρ = −0.68, *P* = .00052, *n* = 48) between SOM and slower/faster-growing bacterial taxa under mouldboard-plough tillage. However, this relationship was absent under cultivator tillage (*P* = .21, *n* = 48). We observed similar trends in CAZyme genes per genome and SOM (Fig. S9) with growth rates and SOM, but since the distribution was highly deviating from Gaussian, these results might not be fully representative. Meanwhile, we did not observe such trends in genome size (Fig. S10). Overall, our results indicated that tillage affected not only the slower/faster-growing bacterial ratio but also influenced how SOM and slower/faster-growing bacteria were correlated.

**Figure 6:**
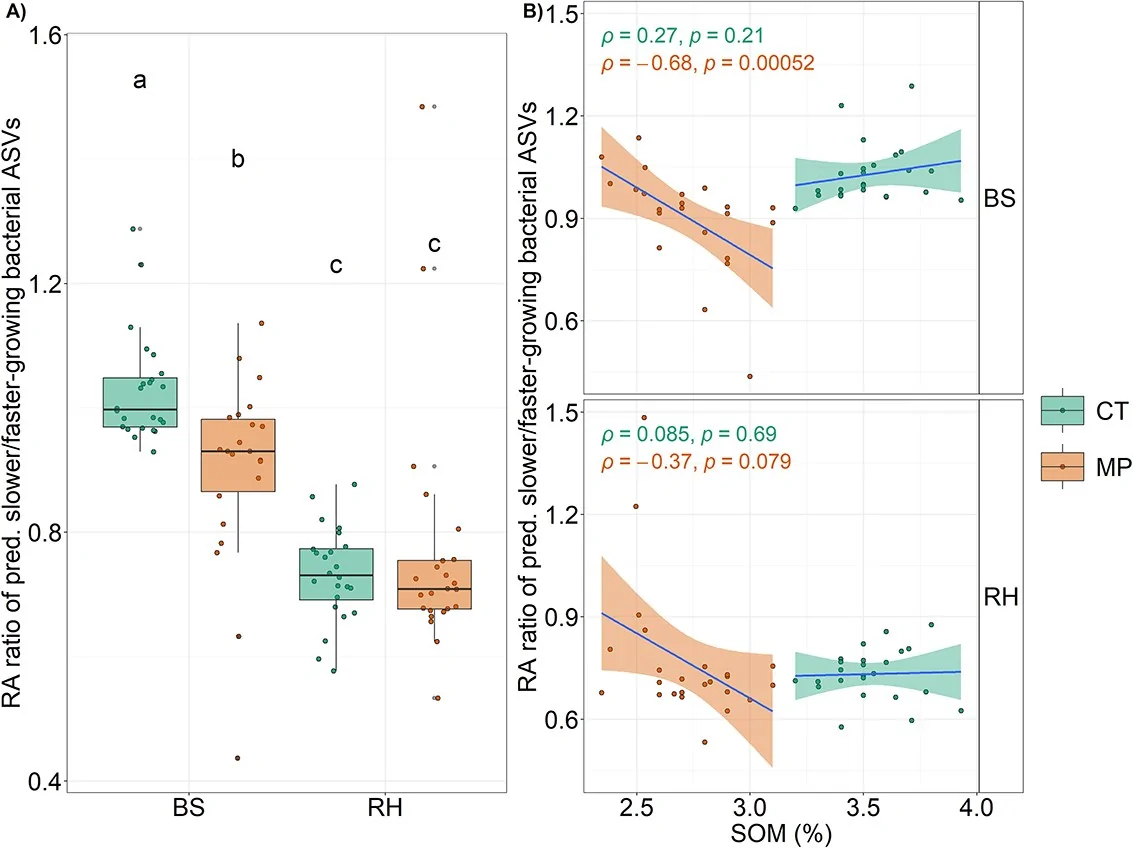
(A) Boxplots showing ratios of relative abundance (RA) for predicted (pred.) slower- and faster-growing bacterial ASVs across soil habitats and tillage practices. We mapped all ASVs in the datasets to GTDB genomes and assigned each ASV a predicted doubling time (if one ASV was mapped to more than one genome, the mean predicted doubling time was used). Then we evaluated the population distribution of predicted doubling time for the population of ASVs with mapped genomes (Fig. S4) and assigned the slower/faster-growing ASVs based on the above/below median pmDTs. As a final step, we calculated their ratio to indicate whether tillage practice or soil microhabitat induced any shifts to those ratios. Statistically significant differences between groups are denoted by different letters above the bars (Dunn’s test with Benjamini-Hochberg correction, *P* < .05, *n* = 24). (B) Correlations between SOM and RA ratios across soil habitats and tillage practices were made on ASVs with the ratio of slower/faster bacterial ASVs (Spearman correlation, *n* = 24).

## Discussion

In the present study, we analyzed data from a long-term field experiment on loess chernozem soil over three growing seasons and showed that cultivator tillage led to higher SOM content over the different years. Lower tillage intensity increased the ratio of slower/faster-growing bacterial taxa in compositional datasets and showed higher SOM content when compared to mouldboard-plough tillage. In addition, tillage intensity influenced how the ratio of slower*/*faster-growing bacteria correlates with SOM: the ratio of slower*/*faster-growing bacteria negatively correlated with the SOM content under mouldboard-plough tillage, while under cultivator tillage, this correlation was absent. Based on these results, we hypothesize that cultivator tillage preserves segregated niches that disconnect the availability of SOM to the microorganisms, when compared to mouldboard-plough tillage, which preserves the mixing of SOM and microorganisms, leading to these different relationships. Thus, future studies should evaluate how *K-* and *r*-strategists contribute to shaping SOM decomposition under different levels of aggregation or homogenization/mixing of SOM and microorganisms.

Previous studies have proposed soil microbial processes as a driver for the benefits of less intensive agricultural practices [2, 32]. We demonstrate that reduced tillage intensity, as a part of conservation agriculture, was associated with an increased *K-/r-*strategist compositional ratio. Since cultivator tillage preserved SOM content with a higher proportion of *K-* to *r-*strategists, our results are in agreement with previously observed insights showing that conservation agricultural practices maintain the SOM content through influencing microbial processes [2, 9, 61]. We speculate that soil inversion during mouldboard-plough tillage disrupted SOM aggregates and relocated and uniformly mixed nutrients and microorganisms. Thereby, *r*-strategist taxa might more easily occupy niches with organic sources under mouldboard-plough tillage, which hypothetically could explain the negative correlation of the *K-/r-*strategist ratio with SOM (Fig. 6B). In addition, increased aeration rates in mouldboard-plough tillage may favor aerobic processes. Meanwhile, we presume that cultivator tillage lacks the effects of mouldboard-plow tillage (i.e. dispersal and niche destruction) and is associated with less compacted and undisturbed soils that disconnect the availability of SOM to *r-*strategists. However, as these processes are happening in the topsoil layers, we would not expect such differences at deeper soil layers. Overall, our results indicate that *r*-strategists thrive in disturbed environments and depend on high-nutrient availability. Thus, the ratio of *K*-strategists (oligotrophs) over *r*-strategists (copiotrophs) in soil microbial communities might indicate the resilience and functionality of soil ecosystems.

While reduced tillage increased the dominance of *K-* over *r*-strategist taxa in bulk soil, this effect was absent in the rhizosphere (Fig. 6). Plants actively exude organic compounds into the rhizosphere, which might have overwritten the selection due to tillage intensity [62]. Specifically, a previous study indicated that plants select for microbial taxa with high growth rates across different phyla, such as Actinobacteriota or Proteobacteria [52], using the same approach to infer growth rates based on codon usage [37]. Here, we confirm that the rhizosphere is a nutrient-rich hotspot, since the *K-/r-*strategist ratio was lower in the rhizosphere (Fig. 6A). We speculate that the carbon input due to the rhizosphere effect [62] may have suppressed the effects of tillage intensity on bacterial lifecycle strategies. In our previous works, we selected flowering time as a sampling time-point, since plants switch during flowering from vegetative to generative growth, where it is presumed that root exudation simultaneously shifts from more N-rich compounds to easily degradable low molecular weight carbon (C)-sources, such as simple sugars and organic acids [63, 64]. Root exudation of organic acids and bioactive substances (e.g. benzoxazinoids in maize) with antimicrobial properties, to regulate potentially phytopathogenic taxa, may have shaped rhizosphere bacterial communities [63], which probably masked the effects of agricultural practice in the rhizosphere.

We used a lifecycle-trait based ecological framework for connecting the effects of tillage intensity on microbial life strategies, for the herein investigated LTE site. While ASV datasets are compositional [65], we cannot show, based on the absence of correlation, that the *K-/r-*strategist ratio was disconnected with SOM content under cultivator tillage (Fig. 6). We consider that these results indicate that segregated environments in cultivator tillage contributed to the higher SOM content, when compared to mouldboard-plough tillage (Fig. 1). Moreover, we analyzed soil/rhizosphere microbial communities sampled once a year across three years. Since the long-term field trial has been managed using the same procedure since 2004, soil communities in the different treatments appear to be well-established and adapted to the given conditions. We performed DNA analysis, which included the examination of DNA from dormant or dead bacteria [66]. Since the turnover rates of relic DNA in soils can be slow [67], our approach provided more of a snapshot of the overall effects of agricultural practices. We consider that, to fully determine whether less intensive tillage leads to copiotroph suppression or oligotroph enrichment, time-series experiments incorporating molecular methods, along with analysis of soil enzymatic functions, are needed. In addition, using meta-transcriptomics or amplicons from extracted RNA could also enable us to understand whether less-intensive tillage reduces the abundance of *r*-strategists or promotes *K-*strategist taxa to generate models for *K-/r-*strategist distribution over time.

The analysis of genome size, CAZyme gene numbers per genome (which are widespread among all domains of life and well known to be directly related to carbohydrate metabolism) [68], and especially growth rates (estimated via codon usage bias) appeared feasible and consistent to estimate general metabolic potential and carbohydrate catabolism, thereby assigning related lifecycle strategies [69, 70]. Apart from the herein examined traits, several authors apply rRNA operon copy numbers for this purpose [69] and make use of the ribosomal RNA copy number database (rrndb, [70]). Both methods seem to be adequate; however, we presume that utilizing codon usage instead of rRNA gene copy numbers might be better suited for lifecycle assignments within the oligotroph/copiotroph continuum for partially or incompletely assembled genomes. Moreover, genome size and maximum growth rate have been suggested as poorly correlated and near-independent dimensions of ecological variation across bacteria [71], in agreement with our results, since genome size did not show the same clear trends as growth rates and CAZyme genes (Fig. 6, Fig. S8, Fig. S9). In contrast, growth rates frequently correlate with the number of 16S rRNA gene copies, which greatly differ among *K-*/*r***-**strategists, with *r-*strategists usually showing a higher number of 16S rRNA gene copies [27]. Since our study relies on compositional 16S rRNA gene abundances, our observations indicate an overall shift, which cannot directly translate into precise numbers of *K-/r-*strategist shifts.

Overall, our findings indicate that reduced tillage is associated with higher SOM and shifts in bacterial community composition, and that these shifts are associated with indirectly inferred lifecycle strategy traits, using a genus-level approach for estimating these traits. Specifically, cultivator tillage positively affected SOM in the herein investigated LTE site, which was highly connected to an increase in the compositional proportion of *K-* over *r-*strategists in soil bacterial communities. These results indicate that lifecycle traits of soil microbes might be related to the enrichment of SOM under less intensive tillage. Thus, shifts in the ratio of *K-/r*-strategists in soil microbial communities might contribute to the sustainability of agricultural solutions; however, it is unclear whether and how *K*-strategists enhance the resilience and functionality of soil ecosystems [72]. Consequently, future studies should also expand to further assessment of soil structure and aggregation along with climatic conditions using either defined communities or deep metagenome sequencing to understand how lifecycle-history traits shape SOM composition in agricultural settings.

## Supplementary Material

Supplementary_materials_ycag251

## Data Availability

Raw sequence data were uploaded to the SRA (https://www.ncbi.nlm.nih.gov/sra) and can be accessed via different BioProject or SRP numbers (Table S4). All data from the long-term field experiment are published at the BonaRes Repository (https://doi.org/10.20387/bonares-w669-gdsd). The custom codes for the analysis are available at the GitHub repository https://github.com/JonKampouris/Synthesis_Three_Years_Bernburg and the Zenodo repository (https://zenodo.org/records/17794467). All analyzed genomes can be retrieved from SRA and GTDB (Table S3).
