# Supplementary material for: Positive effects of reduced tillage intensity on soil organic matter are linked to shifts of K-/r-strategists in soil bacterial community composition
Source: ISME Commun. 2026 Sep 7;6(1):ycag251. doi: 10.1093/ismeco/ycag251 (PMC13637816; doi:10.1093/ismeco/ycag251)
Supplement: Supplementary file 1 [file supplementary_materials_ycag251.zip › SupplementaryMaterial.docx]

**Supplementary Material for**

**Positive effects of reduced tillage intensity on soil organic matter are linked to shifts of *K-/r-*strategists in soil bacterial community composition**

Marie Raab^1#^, Jan Helge Behr^2^, Sotirios Vasileiadis^3^, Günter Neumann^4^, Kornelia Smalla^5^, Rita Grosch^2^, Joerg Geistlinger^1^, Wilfried Rozhon^1^, Doreen Babin^5*^, Ioannis D. Kampouris^5,6,7#^

^1^Anhalt University of Applied Sciences, Department of Agriculture, Ecotrophology and Landscape Development, Bernburg, Germany;

^2^Leibniz Institute of Vegetable and Ornamental Crops (IGZ), Plant-Microbe Systems, Großbeeren, Germany;

^3^University of Thessaly, Department of Biochemistry and Biotechnology, Laboratory of Plant and Environmental Biotechnology, Larissa, Greece;

^4^University of Hohenheim, Institute of Crop Science (340h), Stuttgart, Germany;

^5^Julius Kühn Institute (JKI) - Federal Research Centre for Cultivated Plants, Institute for Epidemiology and Pathogen Diagnostics, Braunschweig, Germany;

^6^Department of Applied Microbial Ecology, Helmholtz Centre for Environmental Research GmbH - UFZ, Leipzig, Germany

^7^German Centre for Integrative Biodiversity Research (iDiv) Halle-Jena-Leipzig, Leipzig, Germany

#These authors contributed equally

*Corresponding author:

Dr. Doreen Babin, Julius Kühn Institute (JKI) – Federal Research Centre for Cultivated Plants, Messeweg 11/12, 38104 Braunschweig, Tel: +49 3946 47 6160, [doreen.babin@julius-kuehn.de](mailto:doreen.babin@julius-kuehn.de)

Running title: *Tillage shifts soil bacterial K-/r ratio*

**Table of Contents**

| **No.** | **Title** | **Pages** |
| --- | --- | --- |
| **Fig. S1** | Q-Q Plot for soil organic matter and total nitrogen | **3** |
| **Fig. S2** | Development of SOM (%) and N (%) from baseline 2009 to analysis period 2019 – 2021 | **4** |
| **Fig. S3** | Rarefaction curves for bacterial communities | **5** |
| **Fig. S4** | Density plots showing the different traits of bacterial genomes | **6** |
| **Fig. S5** | PERMANOVA analysis: full bacterial community and dominant fraction across experimental years and soil habitats | **7** |
| **Fig. S6** | Venn diagram: overlap of MP- and CT-responders on bacterial ASV level across soil habitats by experimental year | **8** |
| **Fig. S7** | Venn diagram: overlap of MP- and CT-responders on bacterial ASV level across experimental years in both soil habitats | **9** |
| **Fig. S8** | Analysis for monotonic and non-monotonic relationships between relative abundance (RA) ratios of slower/faster-growing bacterial ASVs and SOM | **10** |
| **Fig. S9** | Analysis of relative abundance (RA) ratios and their relationship with soil habitats and tillage practices for ASVs with low/high predicted aptitude for carbohydrate metabolism | **11** |
| **Fig. S10** | Analysis of relative abundance (RA) ratios and their relationship with soil habitats and tillage practices for ASVs with below/above predicted genome sizes | **12** |
| **Table S1** | Details of the used genomic datasets | **13** |
| **Table S2** | Linear model results for soil organic matter (SOM) and total nitrogen (N) with tillage, N-fertilization, and year as fixed effects | **13** |
| **Table S3** | Logistic regression for differentially abundant bacterial ASVs | **13** |
| **Table S4** | Information about the genomes that were phylogenetically associated with bacterial ASVs based on sequence alignment | **13** |

**Fig. S1. Q-Q Plots of soil organic matter (SOM (%)) and total nitrogen (N (%)) showing the normality of data distribution.**

**Fig. S2. This analysis focuses on the long-term experiment (LTE) in Bernburg Germany, established in 1992, with the last management changes made in 2004. The first measurement of soil organic matter (SOM) was taken in 2009, which serves as the baseline in this plot. The trend analysis shows the development of SOM and nitrogen (N) content from the baseline to the analysis period 2019** – **2021.**

**Fig. S3. Rarefaction curves of bacterial communities from rhizosphere and bulk soil.**

**Fig. S4.** Density plots showing the different traits of ASVs based on average values from their closely phylogenetically related bacterial genomes based on 97% of sequence homology. **A**) Predicted minimal doubling time (hours; pmDT), **B**) predicted genome size (bp), and **C**) predicted number of genes for carbohydrate active enzymes (CAZyme) per genome for all ASVs. Data were log_10_-transformed to improve visualization. Global alignment was performed with ASVs and 16S rRNA genes from the genome taxonomy database (GTDB). Only genomes that showed at least 97% alignment identity with ASVs were included in the analysis. Genome size was predicted with CheckM2 and was corrected for completeness. Minimal doubling times were inferred from codon usage with gRodon2. CAZyme genes were annotated using the dbCAN3 database. The dashed lines show the median.

**Fig. S5.** PERMANOVA results (Bray-Curtis distance) of the full bacterial community and the dominant fraction (mean of the relative abundances, focusing on the 50 most abundant taxa) across experimental years and soil habitats. Significance levels are indicated by asterisks, with the color coding representing the explained variance. The analysis was conducted for tillage (CT vs. MP), N-fertilization (Ext. vs Int.), and their interaction effects (n = 16). **p* < 0.05, ***p* < 0.01, ****p* < 0.001, **** *p* < 0.0001. **BS: bulk soil, RH: rhizosphere.**

**Fig. S6. Venn diagrams showing the number and relative proportion of positive MP- and CT-responders on bacterial ASV level and their overlap (shared ASVs) between bulk soil (BS) and rhizosphere (RH).**

**Fig. S7. Venn diagrams showing the number and relative proportion of positive MP- and CT-responders on bacterial ASV level and their overlap (shared ASVs) between the experimental years in both soil habitats. BS: bulk soil, RH: rhizosphere.**

**Fig. S8. Monotonic and non-monotonic relationship of ratios of relative abundance (RA) for predicted (pred.) slower- and faster-growing bacterial ASVs across soil habitats and tillage practices. We mapped all ASVs in the datasets to GTDB genomes and assigned each ASV a predicted doubling time (if one ASV was mapped to more than one genome, the mean predicted doubling time was used). Then we evaluated the population distribution of predicted doubling time for the population of ASVs with mapped genomes (Fig. S4) and assigned the slower/faster-growing ASVs based on the below/above median distribution. Here, we performed Spearman correlation for bulk soil (BS) and rhizosphere (RH). In addition, we checked for an unimodal relationship: RA Ratio ~ SOM+SOM^2^. Based on ANOVA test, the polynomial model was slightly but significantly a better fit than the linear model (*p =* 0.024) in the BS.**

**Fig. S9. A)** Boxplots showing ratios or relative abundance (RA) for ASVs with low/high predicted (pred.) aptitude for carbohydrate (carb.) metabolism across soil habitats and tillage practices. We mapped all ASVs in the datasets with genomes from GTDB, and assigned to each ASV the number of predicted CAZyme genes per genome, based on the average values of CAZyme genes per genome of closely phylogenetically related genomes. Then we evaluated the distribution of predicted doubling times for the population of ASVs with mapped genomes (Fig. S4) and assigned the ASVs with below/above median aptitude for carb. metabolism based on the median value of CAZyme genes per genome. In the final step, we calculated their ratio to indicate whether tillage practice or soil habitat induced any shifts to those ratios. Statistically significant differences between groups are denoted by different letters above the bars (Dunn's test with Benjamini-Hochberg correction, *p* < 0.05, n = 24). **B)** Correlations between SOM and RA ratios across soil habitats and tillage practices on ASVs with the ratio of bacterial ASVs with low/high predicted (pred.) aptitude for carbohydrate (carb.) metabolism (Spearman correlation, n = 24). Data from all years were combined.

**Fig. S10.** A) Boxplots showing ratios or relative abundance (RA) for ASVs with predicted (pred.) below and above average genome size across soil habitats and tillage practices. We mapped all ASVs in the datasets with genomes from GTDB, and assigned to each ASV a predicted genome size based on CheckM2 (if one ASV was mapped to more than one genome, the mean genome size was assigned). Then we evaluated the distribution of predicted doubling time for the population of ASVs with mapped genomes (Fig. S4) and assigned the ASVs with below/above median genome size based on the median value, and calculated their ratio to indicate whether tillage practice or soil habitat induced any shifts to those ratios. Statistically significant differences between groups are denoted by different letters above the bars (Dunn's test with Benjamini-Hochberg correction, *p* < 0.05, n = 24). **B)** Correlations between SOM and RA ratios across soil habitats and tillage practices on ASVs with the ratio of bacterial ASVs with below/above median genome size (Spearman correlation, n = 24). Data from all years were combined.

**Table S1. Coefficients inferred from linear models, 95% confidence intervals (CI), and *p*-values inferred from 10,000 bootstraps for soil organic matter and total nitrogen (Value~Tillage intensity*N-fert. intensity*Experimental_Year**

***Please check the excel file SupplementaryMaterial.xlsx***

**Table S2. Differentially abundant bacterial ASVs inferred by logistic regression models of centered log-ratio transformed ASV relative abundance versus tillage intensity (CT vs MP), separately by year and soil habitat. Only ASVs with BH-adjusted p < 0.05 were included. RH: rhizosphere; BS: bulk soil**

***Please check the excel file SupplementaryMaterial.xlsx***

**Table S3. Genomes that phylogenetically associated with bacterial ASVs in the present dataset, with sequence homology similarity above 97%. Several genomes have been mapped to more than one ASV, and several ASVs have been mapped to more than one genome.**

***Please check the excel file SupplementaryMaterial.xlsx***

**Table S4. Details of the herein genomic datasets and their access from sequencing read archive. BS: Bulk soil, RH: rhizosphere**

***Please check the excel file SupplementaryMaterial.xlsx***

**Materials and Methods Section: additional information**

**Sampling**

Soil and maize rhizosphere samples were collected at flowering (EC 53-63) during three growing seasons from 2019 until 2021. Per subplot/replicate, root material from three plants was pooled. First, the roots (0-30 cm depth) with adhering soil were shaken vigorously to remove loosely adhering soil (bulk soil), and the roots were briefly washed with sterile tap water. The root systems with strongly adhering soil of all plants per replicate were cut and homogenously mixed. From this mixed root sample, 5 g was used to recover rhizosphere microbial cells by three 1-min blending steps in a Stomacher 400 Circulator (Seward Ltd, Worthing, UK) each with 15 ml of 0.3% NaCl. Total community DNA was extracted from total rhizosphere pellets using the FastPrep-24 bead-beating system and FastDNA Spin Kit for Soil (MP Biomedicals, Santa Ana, CA, USA) following the manufacturer’s recommendations. DNA samples were further purified with the GeneClean Spin Kit (MP Biomedicals, Santa Ana, CA, USA).

**Physicochemical data analysis**

SOM and total N were analyzed with the Wilcoxon rank-sum test and linear models (LM) using the R functions “lm” and the function “Boot” from the R package “car” 1.1.36 [1].

**Bioinformatics and statistical analysis of amplicon sequencing data**

16S rRNA gene amplicon sequencing

We performed DADA2 (v. 1.34.0) with a workflow for different sequencing runs as recommended [2], where error training and prediction for each sequencing round was performed separately. Classification was performed using a Naïve Bayes classifier trained on the SILVA database v. 138.1 [3]. Sequences classified as archaea, mitochondria, and chloroplasts were excluded. Adequate sequencing depth was estimated using rarefaction (Fig. S3). A total of 4,238,732 amplicon reads was obtained, which resulted in 44,153.5 ± 13,830.8 (mean ± standard deviation) reads per sample. ASV abundance was normalized to the sample with the lowest number of reads (21,701 reads) via rarefaction using the package “vegan” v. 2.7.2, which was repeated 1,000 times, and the average read abundances were calculated.

Differential abundance testing and multivariate tests

For differential abundance analysis of the microbiome, we performed logistic regression (LR) with Benjamini-Hochberg correction in the R programming language for every year separately, using the function “glm” and binomial family of the models. The significance of tillage and N-fertilization intensity on community composition was tested with PERMANOVA based on the package “vegan” v. 2.7.2 [4], using the adonis2 function.

**Retrieving genomes from the Genome Taxonomy Database phylogenetically associated with differentially abundant bacterial ASVs**

ASVs were aligned to the 16S rRNA gene dataset from the GTDB using the USEARCH global alignment tool [5] with a minimum sequence similarity of 97% with the following commands: usearch -usearch_global -id 0.97 -evalue 1e^-6^ -strand both -maxaccepts 0 -maxrejects 0 -threads 35 -blast6out. The genomes, whose 16S rRNA gene sequences were annotated to ASVs, were retrieved from the SRA (sequence read archive, www.ncbi.nlm.nih.gov/sra) using EMBOSS toolsets v.6.6.0 [6] using the following command: datasets download genome accession --filename.

**Estimation of traits associated with *K-*/*r-*strategy in bacterial genomes**

Predicted minimal doubling times (pmDTs) were inferred based on codon usage bias via gRODON2 v. 2.4.0 [7] with the command predictGrowth (mode="partial"). Genome sizes were predicted by CheckM2 v. 1.1.0 [8] with the command: checkm2 predict --input --output-directory --database_path uniref100.KO.1. dmnd --threads 30 --tmpdir –force. Genome sizes were corrected based on available completeness and contamination information: Corrected genome size: Genome Size + Genome Size x (1-Completeness/100) - Genome Size x (Contamination/100).

We fitted linear models (slower/faster-growing bacterial taxa ~ SOM) using the function lm(y~x) and polynomial models (slower/faster-growing bacterial taxa ~ SOM + SOM^2^) using the function lm(y~x+poly(x,2)).

**References**

1.	Bates D et al. Fitting linear mixed-effects models using lme4. *J Stat Softw* 2015;**67**. https://doi.org/10.18637/jss.v067.i01

2.	Callahan BJ et al. DADA2: High-resolution sample inference from Illumina amplicon data. *Nat Methods* 2016;**13**:581–583. https://doi.org/10.1038/nmeth.3869

3.	Quast C et al. The SILVA ribosomal RNA gene database project: Improved data processing and web-based tools. *Nucleic Acids Res* 2013;**41**:590–596. https://doi.org/10.1093/nar/gks1219

4.	Oksanen J et al. vegan: Community Ecology Package. *CRAN: Contributed Packages*. https://vegandevs.github.io/vegan/. (29 January 2025, date last accessed).

5.	Edgar RC. UPARSE: Highly accurate OTU sequences from microbial amplicon reads. *Nat Methods* 2013;**10**:996–998. https://doi.org/10.1038/nmeth.2604

6.	Rice P, Longden L, Bleasby A. EMBOSS: the European Molecular Biology Open Software Suite. *Trends Genet* 2000;**16**:276–277. https://doi.org/10.1016/S0168-9525(00)02024-2

7.	Weissman JL, Hou S, Fuhrman JA. Estimating maximal microbial growth rates from cultures, metagenomes, and single cells via codon usage patterns. *Proc Natl Acad Sci U S A* 2021;**118**:1–10. https://doi.org/10.1073/pnas.2016810118

8.	Chklovski A et al. CheckM2: a rapid, scalable and accurate tool for assessing microbial genome quality using machine learning. *Nature Methods 2023 20:8* 2023;**20**:1203–1212. https://doi.org/10.1038/s41592-023-01940-w
